# How Did I Start and Where Am I Going? From a General Water Transfer Protocol of Inorganic Nanoparticles to the Nanotech Approach to Tackle Cancer

**DOI:** 10.1021/acs.nanolett.5c06139

**Published:** 2026-01-23

**Authors:** Teresa Pellegrino

**Affiliations:** 121451 Italian Institute of Technology, via Morego 30, 16163 Genoa, Italy

*Nano Letters* taught me a master lesson: how important it is to publish protocols that are repeatable and reproducible by the whole community

When I began my career, I discovered the shining world of quantum dots (QDs), during a Master fellowship visit in the group of Prof. Paul Alivisatos at the University of California, Berkeley. Those were the years when the first works of QDs application in bioimaging were published.^1–3^ In the work from the Alivisatos’ group,^1^ for example, different subcellular compartments (the cell nuclei and membrane actin filaments of epithelial cells) were stained with QDs of different sizes, providing evidence of multiple color-emitting QDs recording on cells using a single excitation source. Fascinated by this study, I jumped in a project in which we studied the traces free of nanocrystals, thus not anymore fluorescent, left by tumor cells when seeded on top of a preformed and homogeneous red emitting carpet of QDs.^4^ Interestingly, we were able to correlate the areas free of QDs with the migration and invasiveness of the tumor cells. At the same time, I was also involved in another study which turned out to be my first *Nano Letters* publication as a coauthor.^5^ The work focused on understanding the conformational distribution of DNA molecules attached on the surface of gold nanoparticles (NPs) by gel electrophoresis, a technique borrowed from biotechnology. These topics were so stimulating that I immediately understood I was going to build my career at the interface between materials and bioscience.

During my Italian Ph.D. program, I then spent a visiting period in Munich at the Center for Nanoscience, in the group of Prof. Wolfgang Parak. There, driven by the motivation to contribute to the nanoscience field with my materials-based perspective, I successfully set up a universal protocol to transfer hydrophobically capped NPs from the organic phase (the typical medium in which QDs and magnetic NPs were synthesized) to the aqueous phase, so that these NPs could be used in biological experiments.^6^ The protocol consisted in wrapping an amphiphilic polymer around the NP surface, exploiting an interdigitation process of the hydrophobic tails of the polymer with the alkyl tails of the surfactant molecules passivating the surface of the nanocrystals.

The strength of this work, published in *Nano Letters* with me as first author,^6^ lies indeed in the general applicability of the protocol to different types of NPs, equipping them with a stable coating shell that makes them stable in water and suitable for carboxylic-mediated functionalization with different biomolecules. The high reproducibility of the protocol has made it a reliable and versatile method viable to virtually any inorganic NP core. Examples of water-transferred NPs include magnetic NPs (CoPt_3_ and Fe_2_O_3_), plasmonic NPs, QDs, upconverting NPs, and MOF-based materials.^6–8^ Also, the method worked regardless of particle shape (sphere, rod, cube) and complexity (for example, it could be applied to dimer-like NPs and with multi-subdomain NPs).^9^

This protocol over the years was also implemented on a wide range of polymers, including commercially available ones (e.g., polymaleic anhydride*-alt*-octadecene, polyisobutylene-*alt*-maleic anhydride).^10^ This made it particularly appealing for groups that do not necessarily have the skills or the interest in synthesizing their own polymers. When the protocol was integrated with custom-designed anhydride-based polymers, these were modified with various alkyl chains and/or functional molecules to control polymer intercalation and to introduce chemical moieties exploitable for further chemical linking reaction at the NP surface.^11,12^ For example, small functional molecules (e.g., folic acid, biotin, TAT peptides, DOTA complex, fluorescent dyes, gluthatione)^13–15^ as well as large biomolecules such as antibodies or antibody fragments could be covalently linked at the polymer coating surface.^16,17^

In parallel, I was also involved in another work (also published in *Nano Letters*)^18^ in which we carried out comparative cytotoxic studies with QDs coated with different water stable shells. In that work, we demonstrated that the hydrophobic interdigitated layer of the amphiphilic polymer acts as a more effective barrier to prevent Cd^2+^ release than tiny thiol-bearing molecules, which in turn slows down the kinetics of degradation and cytotoxicity. In addition, the use of inorganic shells (ZnSe or silica) and/or the addition of polyethylene glycol (PEG) molecules was shown to provide colloidal stability to NPs thus preventing NPs precipitation in cell media and reduced toxicity of NPs on cells.

These two latter *Nano Letters* publications,^6,18^ especially since they were authored at the very start of my academic career, have strongly marked my pathway, as they taught me a master lesson: how important it is to publish protocols that are repeatable and reproducible by the whole community. I am proud to see that the polymer-coating protocol developed at that time is still actively used in my group today, alongside newer biocompatible coatings, and still continues to play an important role in my research.

The polymer coating protocol has represented a tool to extend the exploration of biomedical applications not only to QDs but to other classes of materials, thus opening new biomedical fields of applications. Indeed, few years later, I ventured into the field of magnetic NPs and started developing stimuli-responsive drug delivery systems that use magnetic NPs as the central core of those platforms.^19,20^ Within a European project which I was coordinating, 11 research groups intensively worked together under a common goal: to exploit the heat generated by magnetic nanoplatforms under a time-varying magnetic field as both an external and remote stimulus to control drug release at the tumor, while inducing at the same time temperature increase at therapeutic values (43–46 °C) and inducing tumor cell damage in the so-called magnetic hyperthermia treatment (MHT).^21–23^

Over the last 20 years, in the field of magneto-oncotherapy, my work has contributed within the community to address challenges related to the development of new scalable synthesis protocols of magnetic NPs with new types of shape (cubic, Rubik-type cube, start-like) to achieve the highest magneto-heat conversion at the lowest dose and under patient-safe radiofrequency conditions.^24,25^ My group has driven the development of controlled assembly of magnetic NPs, exploring how tailored assembly architectures govern collective magnetic behavior, while driving the design of integrated multimodal platforms that combine therapy and imaging.^19,26^

During all these years, my research work, along with that of a broad nanotechnology community, has explored innovative ways to use magnetic induction for diverse applications, for example, to trigger phase transformations in core–shell nanocrystals,^27^ activate catalytic reactions, and declot blood thrombi^28^ and also to stimulate immune cell responses (for example, by activating T cells and innate immune system macrophages and natural killer cells against tumor cells via mild-MHT).^29–31^ More recently, my group has exploited mild-MHT heat-based approaches to prompt magnetic NP uptake by immune cells, a work that was also submitted as an Italian patent application filed in March 2023 (PT240732).

While early strategies relied on macroscopic temperature increases to activate thermal oncotherapy or drug release, more recent and likely future approaches are increasingly focusing on localized MHT-induced heating.^32–34^ This strategy offers dose-independent MHT effects, reduced material-related toxicity, and minimal interference with imaging modalities such as MRI, which are routinely used in the clinic to monitor tumor progression.

For these approaches, measuring the surface temperature at the NP surface is still challenging, especially in vivo. In a study we published in *Nano Letters*,^35^ my group has developed a molecular probe based on a thermosensitive azo molecule which linked fluoresceine amine at varying distances from the magnetic NP surface via PEG spacers. Upon exposure to the time-varying magnetic field, this molecular probe revealed, with a subnanometer resolution, that the effective temperature at the NPs surface is much higher than the bulk temperature of the solution, and the temperature profile exponentially decays over distance from the surface.

These findings provided initial proof for the design of a new class of nanoscale temperature sensors but also promoted the development of magnetic nanoplatforms for remote and combinatorial drug delivery with distinct release kinetics and without macroscopic heating, minimizing collateral tissue damage. Also, local heat effects can induce cell death processes by alternative mechanisms, such as a lysosomal-cell death pathway or overactivation of autophagy or other catabolic pathways.^36^

The local thermomodulation is a basic concept that can be exploited not only for cancer therapeutics but also for remote neuromodulation,^37^ for triggering a thermomechanical stress process^38^ and perhaps mechanobiology, or for thermo-mediated immune modulation via an immunogenic cell death pathway,^30^ or for controlling mitochondrial energy metabolism.

In this regard, other classes of NPs capable of absorbing remotely applied stimuli and dissipating the absorbed energy as heat, beyond radiofrequencies excitation in the kHz range, would also benefit from investigations of localized heating effects.^39^ For instance, photoresponsive nanosystems include plasmonic NPs, copper-based chalcogenide QDs, semiconducting polymer nanoparticles, and Ag_2_S QDs of various sizes, covering the NIR absorption and emission range from 700–900 nm (first window) to 1000–1300 nm (second window). Likewise, microwave-responsive NPs, including carbon-based NPs, MXenes nanomaterials, and iron oxide or mixed-ferrite magnetic NPs, would profit from the local nanoscale heating at the NPs surface.

My group also carried out research on cation-exchange reactions involving radioisotopes in semiconductor NPs, such as the incorporation of Cu-64 ions into Cd- and Pb-free chalcogenide NPs and Y-91 ions into NaGdF_4_ upconverting NPs,^40,41^ thus establishing a new research direction in radiolabeled nanomaterials. This approach, by exploiting the cation exchange within the nanocrystal volume rather than surface association, enables the delivery of substantially higher radiation doses to tumors while using very low NP doses. It thereby achieves exceptionally high specific activity and will potentially redefine internal radiotherapy for cancer treatment.

While many opportunities are envisioned, significant challenges remain to translate the extensive knowledge on nanomaterials into clinical applications, particularly the need to develop nanoparticle-based therapeutics that are scalable, reproducible, sterilizable, standardized, and clinically effective. Nevertheless, as an Italian proverb says, *the climb is tough, but the view is fantastic.”*

On the 25th anniversary of *Nano Letters*, I look back with deep gratitude to the journal for having helped forge both the nanotools I work with and the scientific pathway I have followed.
